# Tetrafluoroethylene-Propylene Elastomer for Fabrication of Microfluidic Organs-on-Chips Resistant to Drug Absorption

**DOI:** 10.3390/mi10110793

**Published:** 2019-11-19

**Authors:** Emi Sano, Chihiro Mori, Naoki Matsuoka, Yuka Ozaki, Keisuke Yagi, Aya Wada, Koichi Tashima, Shinsuke Yamasaki, Kana Tanabe, Kayo Yano, Yu-suke Torisawa

**Affiliations:** 1 Department of Micro Engineering, Kyoto University, Kyoto 615-8540, Japan; e.sano1017@gmail.com (E.S.); mori.chihiro.5u@kyoto-u.ac.jp (C.M.); ozaki.yuka.6z@kyoto-u.ac.jp (Y.O.); yano.kayo.7z@kyoto-u.ac.jp (K.Y.); 2 AGC Inc, Tokyo 100-8405, Japan; naoki-matsuoka@agc.com (N.M.); keisuke-yagi@agc.com (K.Y.); aya-wada@agc.com (A.W.);; 3 Hakubi Center for Advanced Research, Kyoto University, Kyoto 615-8540, Japan

**Keywords:** organs-on-chips, microfluidics, drug absorption, fluoroelastomer

## Abstract

Organs-on-chips are microfluidic devices typically fabricated from polydimethylsiloxane (PDMS). Since PDMS has many attractive properties including high optical clarity and compliance, PDMS is very useful for cell culture applications; however, PDMS possesses a significant drawback in that small hydrophobic molecules are strongly absorbed. This drawback hinders widespread use of PDMS-based devices for drug discovery and development. Here, we describe a microfluidic cell culture system made of a tetrafluoroethylene-propylene (FEPM) elastomer. We demonstrated that FEPM does not absorb small hydrophobic compounds including rhodamine B and three types of drugs, nifedipine, coumarin, and Bay K8644, whereas PDMS absorbs them strongly. The device consists of two FEPM layers of microchannels separated by a thin collagen vitrigel membrane. Since FEPM is flexible and biocompatible, this microfluidic device can be used to culture cells while applying mechanical strain. When human umbilical vein endothelial cells (HUVECs) were subjected to cyclic strain (~10%) for 4 h in this device, HUVECs reoriented and aligned perpendicularly in response to the cyclic stretch. Moreover, we demonstrated that this device can be used to replicate the epithelial–endothelial interface as well as to provide physiological mechanical strain and fluid flow. This method offers a robust platform to produce organs-on-chips for drug discovery and development.

## 1. Introduction

Predicting drug efficacy and toxicity before clinical trials is crucial for the drug discovery and development processes [1,2,3]. In vitro systems that can reliably predict responses to drugs in humans could be a powerful platform to test drugs and to discover new therapeutics. The organs-on-chips technology, specifically, has proven useful for studying physiological mechanisms and pharmacological modulation as well as for developing disease models [4,5,6,7,8]. By mimicking natural tissue architecture and microenvironmental chemical and physical cues within microfluidic devices, organs-on-chips enable the reconstitution of complex organ-level functionality that cannot be recapitulated with conventional culture systems. Since numerous of the physiological microenvironments inside the human body are microfluidic in nature (e.g., the pulmonary system, liver sinusoids, and vascular networks), the use of microfluidic devices facilitates engineering of cellular microenvironments [9,10,11,12]. Microfluidic devices allow for control of local chemical gradients and dynamic mechanical forces which govern the development and function of organs [13,14]. Microfluidic organs-on-chips demonstrated that mechanical forces are important to drive cellular differentiation and function and to faithfully recapitulate human organ-level physiology and pathophysiology; e.g., in lung [15,16,17], gut [18,19], kidney [20,21,22], and cancer models [23]. Thus, these microfluidic devices mimicking the mechanical microenvironment of living tissues have great potential to predict human responses to drugs and serve as an alternative to animal models.

Polydimethylsiloxane (PDMS) is commonly used to fabricate microfluidic devices because it is easy to use, biocompatible, highly gas permeable, optically clear, and flexible [24,25]. Using soft lithography, various types of culture devices have been developed and widely used as tools to study basic and applied scientific research. Although PDMS devices are very useful, one serious drawback is that small hydrophobic molecules are strongly absorbed into PDMS surrounding microchannels [25,26,27,28,29,30,31]. This limitation is critical for the development of in vitro systems to test drugs because many pharmaceutical compounds are small hydrophobic molecules. It has been demonstrated that the use of PDMS-based devices significantly reduces concentrations and the effectiveness of drugs including anticancer drugs due to absorption into PDMS microchannels [29,30]. Although several types of elastomers and coating methods have been developed to reduce absorption of small hydrophobic molecules [32,33,34,35], they have not been widely adopted and there is still no elastic device which resists absorption of small hydrophobic drugs. To overcome this challenge, we explored the possibility of using a fluoroelastomer which is known to have excellent chemical resistance as an alternative material [36,37,38]. We developed a compartmentalized microfluidic device [39] consisting of two layers of microchannels made from a tetrafluoroethylene-propylene (FEPM) elastomer and a thin collagen vitrigel membrane. Furthermore, we demonstrated that drug absorption by the FEPM elastomer is comparable with that by standard cell culture plates made from polystyrene. This FEPM-based microfluidic device can be used to culture cells while applying physiologically relevant mechanical signals.

## 2. Materials and Methods

### 2.1. Device Fabrication

**Figure 1:**
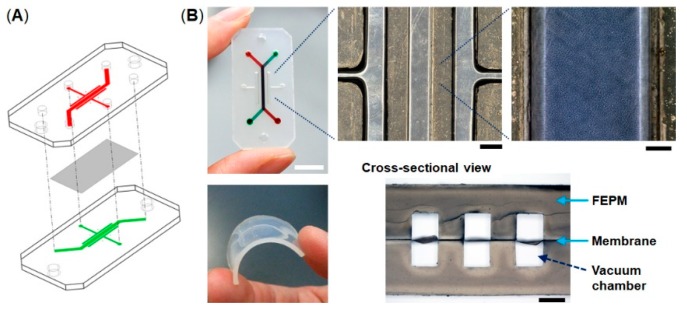
Compartmentalized microfluidic device fabricated from tetrafluoroethylene-propylene (FEPM) elastomer. (**A**) Schematic illustration of the microfluidic device. Two FEPM channel layers are separated by a collagen vitrigel membrane. (**B**) Photographic images of the device and the FEPM channel layer (bottom left). A cross-sectional view shows the top and bottom channels (1 mm in width and 1 mm in height) with the collagen vitrigel membrane (10 µm thick) in-between (bottom right). The gaps between each channel are 1 mm. Cells are cultured in the central channel and mechanical strain is exerted by applying vacuum to all the side vacuum chambers. Scale bar, 10, 1, and 0.2 mm for low, medium, and high magnification views, respectively.

The microfluidic devices consist of two layers of microchannels separated by a thin collagen vitrigel membrane (Figure 1). The microchannel layers were fabricated from tetrafluoroethylene-propylene (FEPM) compounds (AFLAS, AGC Inc., Tokyo, Japan) at a ratio of 10:4 base to vulcanizing agents at 60 °C on a two-roll mill (φ8′’ × 18′’ test roll machine for chemical machine design and production, Yamatetsu Machinery Inc., Tokyo, Japan). Each layer was formed by compression molding with a custom-designed hard-chrome-plated two-layer mold on a 200-ton vacuum compression molding machine (TYC-V-2RT, Tung Yu Hydraulic Machinery Co., Ltd., Nantou, Taiwan), press-curing at 160 °C for 30 min, and post-curing at 200 °C for 2 h in an oven (DH612, Yamato Scientific Co., Ltd., Tokyo, Japan) [37,38]. The cross-sectional size of microchannels is 1 mm in width × 1 mm in height and the gaps between each microchannel are 1 mm. A 10-µm-thick collagen vitrigel membrane (VIT-C001, AGC Techno Glass Co., Ltd., Shizuoka, Japan) was placed between two channel layers. The FEPM layers and the collagen vitrigel membrane were bonded together for 5 s at room temperature by a self-adhesion system that was generated during the formation of each FEPM layer, allowing the channel layers to be assembled without the use of glue. The bonding strength of the FEPM device containing the membrane was measured using a digital spring scale (PS-01, Dr. meter, London, England) and a vertical drill guide (DS-70, SK11, Hyogo, Japan) by pulling the FEPM layer vertically until it detached (n = 5). The dimensions of the FEPM layer were 20 mm (width) × 40 mm (length) × 2 mm (thickness) (Figure S1). The FEPM microfluidic devices were sterilized by placing them under UV light for 2 h prior to cell culture.

To analyze the absorption of PDMS, slabs and microfluidic devices were fabricated from polydimetyisiloxane (Sylgard 184, Dow Corning, Midland, TX, USA) at a ratio of 10:1 base to curing agents using standard soft lithographic techniques [40]. The PDMS microfluidic device consists of a single microchannel similar to the FEPM device without side chambers. The microchannel layers were produced by casting PDMS prepolymer against the mold composted of SU-8 2150 (MicroChem, Westborough, MA, USA) patterns formed on a silicon wafer. PDMS was cured at 60 °C overnight. The channel layers were formed against the relief channel feature 300 µm in height and 1 mm in width. Two channel layers were bonded using oxygen plasma treatment (Covance MP, Femto Science, Hwaseong, Korea) at 500 mTorr pressure and 40 W power for 30 s followed by curing at 60 °C for 2 h.

### 2.2. Analysis of Absorption

To analyze absorption of a fluorescent dye, the inlet and outlet of the microfluidic devices were connected with silicon tubes (i.d. 1 mm, #1018-03, ARAM, Osaka, Japan) and a solution of 10 µM rhodamine B (Nacalai Tesque, Kyoto, Japan) in phosphate-buffered saline (PBS, Nacalai Tesque, Kyoto, Japan) was flowed through the microchannels in the devices using a syringe pump (NE-1000, New Era Pump Systems Inc, Farmingdale, NY, USA) at a flow rate of 1 µL·min^−1^ for up to 24 h. After washing with 10 mL of PBS, fluorescence images were obtained using an inverted microscope (IX-83, Olympus, Tokyo, Japan) with the 4× objective and a microscope digital camera (DP80, Olympus) and were analyzed with cellSens software (Olympus). We used the same exposure time (30 ms) to obtain fluorescent images. The fluorescent light source was a 100 W mercury lamp.

To evaluate drug absorption, slabs (2 mm thick × 6 mm in diameter) made from PDMS and FEPM were placed in polystyrene (PS) 96-well culture plates (AGC Techno Glass). Drug concentrations were determined by high-performance liquid chromatography (HPLC, Shimadzu Corporation, Kyoto, Japan) using a solvent delivery system equipped with an auto sampler. Three types of drugs, nifedipine (N0528, Tokyo Chemical Industry, Tokyo, Japan), Bay K8644 (B112 Sigma-Aldrich, St. Louis, MO, USA), and coumarin (031-16562, Fujifilm Wako Chemicals, Osaka, Japan), were used to test drug absorption. 1 µM of each drug in PBS was incubated for 0.5, 1, 2, 3, 6, and 24 h in PS 96-well plates with or without PDMS or FEPM slabs at 37 °C. An ultraviolet–visible (UV–Vis) absorbance detector (SPD-M20A, Shimadzu Corporation) was used to monitor the UV absorption maximum of the compounds under investigation. A ZORBAX 300SB-C18 column (4.6 × 150 mm, Agilent technologies, Santa Clara, CA, USA) was applied with a stationary phase of C18 (5 µm particles). As mobile phase, acetonitrile was used at 40–70% (depending on the compound) and 0.1% trifluoroacetic acid. Flow speed was 1.0 mL·min^−1^ and the injection volume was 100 µL. Recording and processing of data was performed using LabSolutions software (Shimadzu Corporation). In every experiment, a calibration curve was included to validate the system and the linearity of the compound UV absorption response.

### 2.3. Cell Culture

Green fluorescent protein expressing human umbilical vein endothelial cells (GFP-HUVECs, #cAP-0001GFP, Angio-Proteomie, Boston, MA, USA) and human umbilical vein endothelial cells (HUVECs, #C2517A, Lonza, Basel, Switzerland) were cultured in endothelial cell growth medium (EGM-2, Lonza) with 100 U·ml^−1^ penicillin and 100 U·ml^−1^ streptomycin (P/S, Nalacai Tesque, Kyoto, Japan) and cells between passage 5 and 7 were used for experiments. Caco-2 human intestinal epithelial cells (C2BBe1 clone of Caco-2 human colorectal adenocarcinoma cell line, #CRL-2102, ATCC, Manassas, VA, USA) were cultured in Dulbecco’s Modified Eagle’s Medium (DMEM 08458-45, Nacalai Tesque, Kyoto, Japan) containing 10% fetal bovine serum (FBS F7524, Sigma-Aldrich, St. Louis, MO, USA), MEM non-essential amino acid (Gibco, Dun Laoghaire, Ireland), 100 U·ml^−1^ penicillin, and 100 U·ml^−1^ streptomycin. Cells were cultured in an incubator at 37 °C and 5% CO_2_.

### 2.4. Microfluidic Cell Culture

To culture HUVECs, the collagen vitrigel membranes in the FEPM devices were coated with fibronectin (100 µg·mL^−1^) for 1 h prior to cell seeding. HUVECs (2.5 × 10^4^ cells per 20 µL) were introduced into the top channel and incubated at 37 °C for 60 min to allow the cells to adhere to the membrane. The attached cells were then perfused with culture medium using a micro peristaltic pump (RP-TXP5F, Aquatech Co., Ltd., Osaka, Japan) at a flow rate of 10 µL·min^−1^ (0.008 dyne·cm^−2^) [41]. To examine the effect of cyclic strain, HUVECs cultured in the devices were stretched with 5%–10% strain at a frequency of 1 Hz (sinusoidal waveform) for 4 h by applying vacuum (30–40 kPa) to the side vacuum chambers of the devices using a vacuum controller (FX-500, Flexcell International Corporation, Burlington, VT, USA). Tubing (i.d. 1/16 inch, Tygon LMT-55, Saint-Gobain, La Defense, France) was connected from the vacuum source to the side chambers using needles (16G, Musashi Engineering, Inc., Osaka, Japan). The mechanical strain was evaluated by measuring the displacement between two points before and after stretching [15].

To form the epithelial-endothelial interface, the collagen vitrigel membranes in the FEPM devices were coated with Matrigel (400 µg·mL^−1^, BD Biosciences, Franklin Lakes, NJ, USA) and rat type I collagen (50 µg·mL^−1^, Gibco) for 2 h prior to cell seeding. Caco-2 cells (2.5 × 10^4^ cells per 20 µL) were introduced into the top channel and incubated at 37 °C for 60 min to allow the cells to adhere to the membrane. The attached cells were then perfused with culture medium using a micro peristaltic pump at a flow rate of 10 µL·min^−1^. After 4 days in culture to form a confluent monolayer of Caco-2 cells, the bottom side of the collagen vitrigel membrane was coated with fibronectin (100 µg·mL^−1^) for 30 min by injecting the solution into the bottom channel. After washing with culture medium, HUVECs (2.5 × 10^4^ cells per 20 µL) were introduced into the bottom channel and then the device was inverted and incubated at 37 °C for 30 min to make HUVECs adhere onto the bottom side of the membrane. The attached cells were then perfused at 10 µL·min^−1^ with DMEM and EGM-2 in the top and bottom channels, respectively. The devices were cultured for 3 more days to form a confluent monolayer of HUVECs.

### 2.5. Analysis of Cellular Viability

Cellular viability was assessed by fluorescence microscopy imaging. After 7 days in culture, the channels of the FEPM device were washed with PBS and then the upper channel was filled with a mixture solution containing Calcein-AM (2 µM in PBS) and ethidium homodimer-3 (4 µM in PBS) and incubated for 30 min according to standard protocol (Live/Dead Cell Staining Kit 2, PromoCell, Heidelberg, Germany). To test drug effect, cells were treated with 10 µg·mL^-1^ mitomycin C (Nacalai Tesque) for 24 h. HUVECs were cultured in the FEPM devices and in 96-well culture plates (#353072, Falcon, Corning, NY, USA) for 24 h prior to drug treatment. After 24 h treatment of mitomycin C, the devices and plates were washed with PBS and then were filled with a mixture solution containing calcein-AM and ethidium homodimer-3. Cellular viability was quantified by the percentage of calcein-AM-labeled cells (green) averaged over 3 different observation areas of the device from 3 independent experiments. All cellular images were taken using a microscope digital camera (DP80, Olympus) mounted on an inverted microscope (IX-83, Olympus) with the 4× and 10× objectives.

### 2.6. Analysis of Cellular Alignment

Morphological responses of cells to cyclic strain were quantified using angle of orientation [15,42]. The orientation angle is defined as the angle between the major axis of the best-fit ellipse around a cell and the axis of cyclic strain. The images were obtained by phase-contrast microscopy and analyzed with cellSens software (Olympus) to measure the angle of orientation. The data was analyzed statistically by the F-test at a 99% confidence level.

### 2.7. Immunofluorescence

Cells cultured in the FEPM devices were fixed with 4% paraformaldehyde (PFA, Nacalai Tesque, Kyoto, Japan) for 20 min, washed with PBS, and permeabilized with 0.1% Triton X-100 (Sigma-Aldrich, St. Louis, USA) for 15 min. The cells were then incubated with blocking buffer containing 1% bovine serum albumin (BSA, Sigma-Aldrich) for 30 min at room temperature and incubated with antibodies directed against vascular endothelial (VE)-cadherin (Abcam, ab33168, dilution 1:200, Cambridge, UK) or Claudin (Abcam, ab15098, dilution 1:200) overnight at 4 °C, followed by PBS washes. Subsequently, Alexa 568-conjugated secondary antibody (Abcam, ab175471, dilution 1:500) was introduced into the channels and incubated for 1 h at room temperature. The cells were co-stained with 4’,6-diamidino-2-phenylindole (DAPI, Invitrogen, D1306, Waltham, USA). Cross-sectional images were obtained at 2 µm intervals in the vertical direction using a confocal microscopy (FluoView FV1000 confocal, Olympus, Tokyo, Japan) with the 10× objective.

### 2.8. Statistics

Results are reported as mean ± standard deviation. The F-test was performed to analyze variance for two samples. The Levene test was performed for three samples to confirm that the variances are homogeneous. We confirmed that the data were normally distributed. The statistical significance of variance across groups was assessed by one-way analysis of variance (ANOVA) with a post-hoc analysis using the Bonferroni test. Significance level of P < 0.01 is denoted in graphs by an asterisk (*). Representative results from at least three independent biological replicates are shown.

## 3. Results

### 3.1. Characterization of Tetrafluoroethylene-Propylene (FEPM) Elastomer Devices

**Figure 2:**
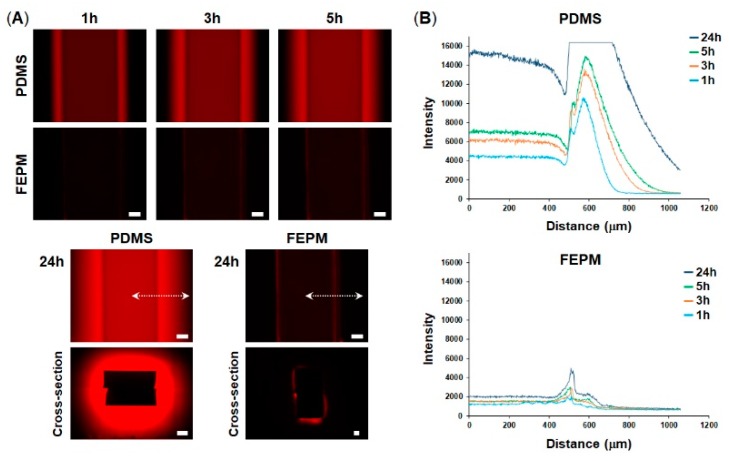
Absorption of rhodamine B into polydimethylsiloxane (PDMS) and FEPM. (**A**) Fluorescent images of rhodamine B absorbed into the PDMS and FEPM channels after 1, 3, 5, 24-h perfusion of rhodamine B solution. Bottom, cross-sectional images of the PDMS and FEPM channels after 24 h perfusion of rhodamine B solution. Scale bars, 200 µm. (**B**) Line profiles of the absorption of rhodamine B inside the walls of the PDMS and FEPM channels. The X-axis shows distance from the center of the channel (indicated by white dotted lines). (n = 3).

To explore whether our microfluidic devices fabricated from FEPM could prevent drug absorption, we compared absorption of a fluorescent dye into microchannels made either of FEPM or PDMS. A solution containing rhodamine B, which is a small hydrophobic fluorophore, was perfused into the microchannels and fluorescence intensity of the microchannels was monitored for 24 h (Figure 2). When the rhodamine B solution was placed in the FEPM and PDMS channels, fluorescence intensity of the FEPM channel was almost same as that of the PDMS channel (Figure S2), demonstrating that the FEPM devices can be used to evaluate this fluorophore. The fluorescence intensity of the PDMS microchannel gradually increased over time and the bright area also gradually spread out, indicating that the PDMS microchannel continuously soaked up the fluorophore. On the other hand, the fluorescence intensity of the FEPM microchannel almost did not change for 24 h and only weak fluorescence signals were observed due to adsorption of the fluorescent dye on the channel wall, demonstrating that the FEPM microchannel is resistant to absorption of this fluorophore. The cross-sectional images of the microchannels clearly showed that the fluorescent compound was absorbed inside the walls of the PDMS microchannel, whereas it was only observed on the channel surface and did not absorb into the FEPM microchannel. These results demonstrate that the FEPM devices are resistant to absorption of rhodamine B.

**Figure 3:**
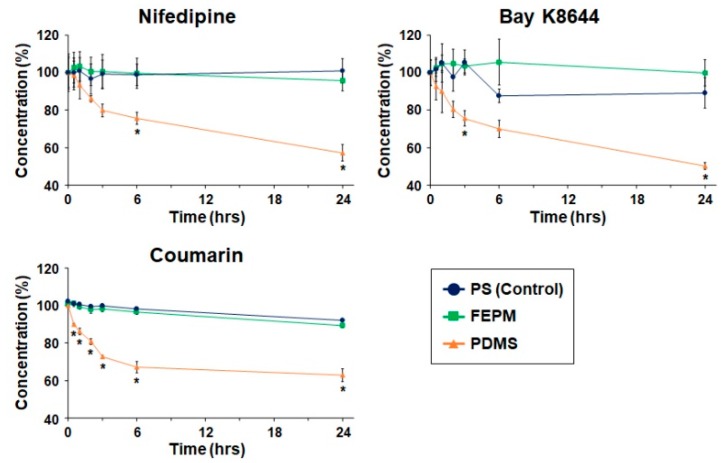
Time-course analysis of drug absorption into PDMS, FEPM, and polystyrene (PS) culture plates (control). Drug concentrations after 0.5, 1, 2, 3, 6, and 24-h incubation of nifedipine, Bay K8644, and coumarin evaluated by high-performance liquid chromatography (HPLC, n = 3, * *p* < 0.01).

We then analyzed absorption of drugs quantitatively by measuring drug concentrations using HPLC. Three types of drugs, nifedipine, Bay K8644, and coumarin, were used to evaluate absorption by FEPM and analyzed by comparing with that by PDMS and polystyrene (PS) 96-well plates (Figure 3). These three drugs are small hydrophobic molecules known to absorb into PDMS [27,31]. The concentrations of these three drugs after incubation with PDMS decreased over time and reached almost half of the initial concentrations after 24 h of incubation. Importantly, the concentrations of all three drugs retrieved from wells containing PDMS slabs were significantly lower than those retrieved from standard PS wells after 24 h of incubation. On the other hand, the concentrations of all three drugs after incubation with FEPM did not change for 24 h. There was no significant difference in the residual concentrations of three drugs between the FEPM and PS well plates. The concentrations of Bay K8644 retrieved from wells containing FEPM slabs were even slightly higher than that in PS well plates over 6 h of incubation. These data clearly demonstrate that the FEPM elastomer is resistant to absorption of small hydrophobic drugs and is comparable with the standard cell-culture plates.

### 3.2. Cell Culture within FEPM Devices

We utilized the FEPM elastomer to construct compartmentalized microfluidic devices as a cell culture system [39]. The device consists of two channel layers separated by a thin collagen vitrigel membrane. We first evaluated bond strength between the FEPM layers with the collagen vitrigel membrane in-between and obtained the minimum bond strength of 105 kPa (average value is 117 ± 13 kPa, n = 5). This bond strength is sufficient to maintain solutions inside channels without leakage during cyclic stretching although the value is lower than that of PDMS layers using plasma treatment (ca. 200 kPa) [43].

**Figure 4:**
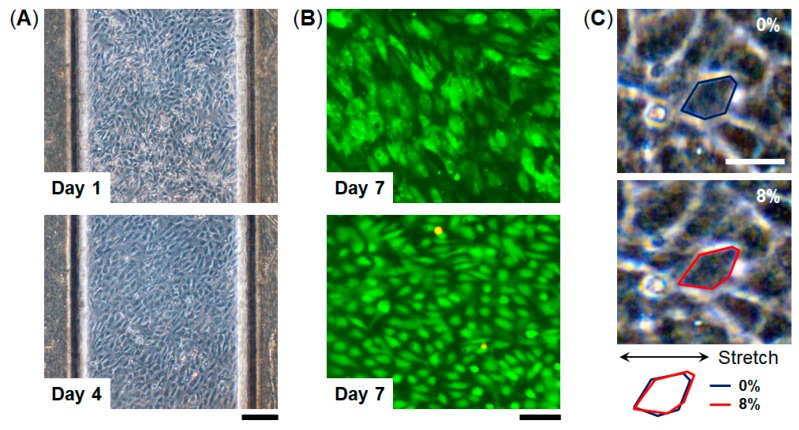
Microfluidic cell culture within FEPM devices. (**A**) Phase contrast micrographs of human umbilical vein endothelial cells (HUVECs) cultured within the device for 1 and 4 days. Scale bar, 200 µm. (**B**) Fluorescent images of green fluorescent protein (GFP)-expressing HUVECs cultured in the device for 7 days. Images were taken under different conditions. Top, green florescence shows GFP of the cells in the device. Bottom, green and red florescence represents live and dead cells, respectively. Scale bar, 100 µm. (n = 3) (**C**) Phase contrast images of HUVECs cultured within the FEPM device in the absence (top) and presence (bottom) of mechanical strain (8%) exerted by applying vacuum to the side chambers. Blue and red outlines indicate the shape of a single cell before (blue) and after (red) mechanical strain application. Scale bar, 50 µm.

To examine cell culture in this device, we used human umbilical vascular endothelial cells (HUVECs) which are commonly used as a representative endothelial cell population. HUVECs were seeded into the top channel of the device and were cultured on the collagen vitrigel membrane under continuous medium perfusion for 7 days (Figure 4). HUVECs formed a monolayer cell sheet after 4 days in culture on-chip and maintained the GFP expression for 7 days. Importantly, almost all the cells were maintained viable for 7 days (viability = 98%, n = 3), indicating that the FEPM-based microfluidic devices can be used to culture cells and monitor cellular responses. We confirmed that HUVECs cultured in the devices are similar to those cultured in cell-culture plates (Figure S3). The cells were able to be cultured for at least 7 days within the microchannels without any leakage, suggesting that the bond strength of the FEPM layers is sufficient to culture cells under fluidic flow for 7 days. We then used this FEPM-based microfluidic device to produce cyclic stretching to mimic physiological movement inside the body. The level of applied mechanical strain ranged from 5% to 10% to match physiological levels of strain [44]. Membrane stretching causes distortion of cell shape as observed by increases in the length of adherent cells in the direction of applied tension (Figure 4C and Video S1). Microscopic analysis of cell shape confirmed that about 8% strain was exerted on the cells by applying suction to the vacuum chambers in the device. Thus, this device can be used to culture cells while applying physiological mechanical strain.

**Figure 5:**
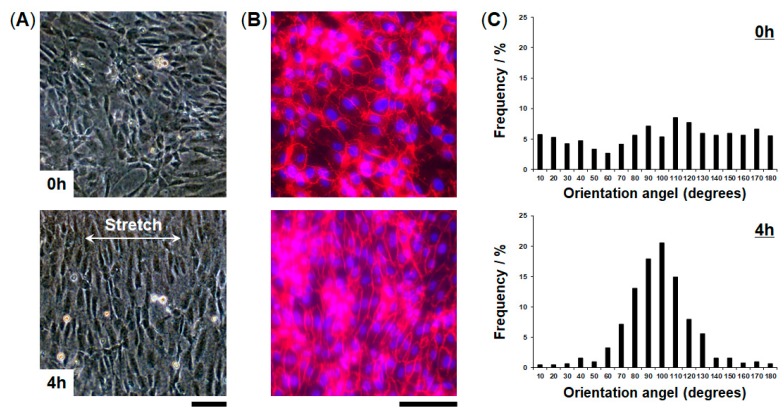
Alignment of vascular endothelial cells induced by application of cyclic strain. (**A**) Phase contrast micrographs of HUVECs cultured in the FEPM device before (top) and after (bottom) 4-h cyclic stretch with ~10% strain at a frequency of 1 Hz. (**B**) Fluorescent images of HUVECs cultured in the device in the absence (top) and presence (bottom) of cyclic strain. HUVECs were stained for vascular endothelial (VE)-cadherin (red) and 4’,6-diamidino-2-phenylindole (DAPI) (blue). Scale bars, 100 µm. (**C**) Histograms of cell orientation before (top) and after (bottom) 4 h of cyclic stretching. (n = 3).

We evaluated cellular responses induced by mechanical strain. HUVECs cultured in the FEPM devices were exposed to physiological cyclic strain (5–10%, 1Hz) for 4 h (Figure 5). Application of physiological cyclic strain induced the endothelial cells to reorient and align perpendicularly. Immunofluorescence analysis demonstrated that HUVECs elongated and aligned in the direction perpendicular to the applied strain (Figure 5B). Quantitative analysis showed a uniform distribution of the orientation angle in the absence of cyclic strain (Figure 5C). When the cells were exposed to cyclic strain for 4 h, the distribution of the orientation angle was biased toward 90°, as compared to control without strain (F-test, *p* < 0.01). Over 80% of the cells exhibited alignment with orientation angles between 60° and 120°. These results demonstrate that this device can be used to create physiological levels of mechanical strain and to monitor cellular responses to mechanical signals.

### 3.3. Engineering of FEPM-Based Organs-on-Chips

To model complex physiological functions of epithelia, organs-on-chips typically recreate tissue-tissue interfaces separated by thin porous membranes coated with extracellular matrix. To explore whether our microfluidic device can be used to replicate tissue-tissue interfaces, we carried out microfluidic culture to form the interface between intestinal epithelial cells and vascular endothelial cells using HUVECs and Caco-2 cells which are commonly used as an intestinal epithelial model [18].

**Figure 6:**
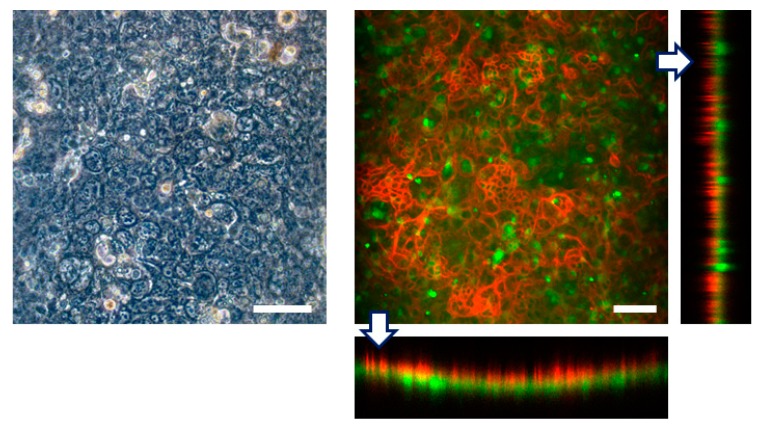
Engineering of tissue-tissue interfaces within FEPM devices. Human intestinal epithelial (Caco-2) cells are cultured on the upper surface of a collagen vitrigel membrane with vascular endothelial cells (HUVECs). Optical and confocal fluorescence micrographs of co-culture of Caco-2 cells stained for claudin (red) and GFP-HUVECs (green). Cross-sectional views (bottom and right) show the formation of monolayers of epithelium and endothelium on both sides of the membrane. Scale bars, 100 µm. (n = 3).

First we seeded Caco-2 cells into the top channel and cultured on the top side of the collagen vitrigel membrane within the FEPM device under continuous medium perfusion. After 4 days in culture to form a monolayer of epithelial cells, HUVECs were seeded into the bottom channel and cultured on the bottom side of the membrane for 3 more days. Immunofluorescence analysis confirmed that confluent cell layers formed on the both sides of the membrane; Caco-2 cells formed a confluent epithelium layer with well-developed tight junctions and HUVECs formed a confluent endothelium (Figure 6). Because the collagen vitrigel membrane swells when exposed to liquid, a curvature of the membrane was observed; however, confluent cell layers were able to form on the both side of the membrane. Thus, this device allows us to replicate a tissue–tissue interface between epithelium and vascular endothelium. These results demonstrate that the FEPM-based microfluidic devices could be used to build organs-on-chips for drug discovery and development.

## 4. Discussion

Organs-on-chips produce tissue-level functionality not possible with standard in vitro culture methods by recapitulating tissue–tissue interfaces and physicochemical microenvironments. This technology has great potential to facilitate drug discovery and development and to replace animal models for drug testing. However, one significant drawback current organs-on-chips have is that devices are mostly made from PDMS which strongly absorbs small hydrophobic molecules. Since drug absorption into PDMS causes a reduction of drug concentrations and pharmacological activities, PDMS-based devices cannot widely be used as tools for drug discovery and development.

In this study, we investigated the possibility of using a tetrafluoroethylene-propylene (FEPM) elastomer to construct microfluidic organs-on-chips for drug testing. We demonstrated that the FEPM elastomer is resistant to absorption of three types of small hydrophobic drugs, whereas PDMS significantly absorbed these drugs, indicating that PDMS is not suitable for constructing devices to test drugs as previously reported [29,30]. By analyzing drug concentrations after 24-h incubation with FEPM, we found that the residual concentrations of these small hydrophobic drugs were almost the same level as those in standard culture plates, demonstrating that the FEPM elastomer is comparable to standard polystyrene culture plates in terms of absorption of small hydrophobic drugs.

We then developed compartmentalized microfluidic devices using the FEPM elastomer. The device comprised two FEPM channel layers separated by a collagen vitrigel membrane which is made from natural extracellular matrix (ECM). The cellular images demonstrated that the FEPM layers are optically clear and can be used for fluorescence imaging. The FEPM elastomer is hydrophobic and has Young’s modulus of 0.8 MPa which is similar to PDMS [45]. Thus, the FEPM-based device can produce cyclic stretching of the ECM membrane with physiological levels of strain (~10%) by applying cyclic vacuum to the side chambers. We confirmed that there was no leakage between each layer and the collagen vitrigel membrane during cell culture with cyclic stretching. We also confirmed that this device can be used to culture cells and maintain cellular viability with continuous perfusion of culture medium for at least 7 days. By seeding cells into the both sides of the microchannels, the epithelial-endothelial interface can be formed in this device, suggesting that this device can be used to construct organs-on-chips. As an example, we demonstrated that an intestinal epithelial model can be constructed by replicating the interface between intestinal epithelial cells and vascular endothelial cells using Caco-2 cells and HUVECs. Furthermore, this device permitted imaging of cellular responses to environmental signals such as mechanical forces in real-time. When HUVECs were subjected to physiological cyclic strain (5–10%) for 4 h, the cells exhibited elongation and alignment on the ECM membrane in response to the cyclic strain. Moreover, we confirmed that this device can be used to test cytotoxic effect of mitomycin C (Figure S4). After 24 h treatment of mitomycin C (10 µg·mL^−1^), there was no significant difference in the number of live and dead cells between the device culture and the conventional plate culture. Thus, this device provides a way to recapitulate cellular microenvironment including cyclic stretching and fluid flow for drug testing.

Since the FEPM elastomer is biocompatible, flexible, and optically clear, it has the potential to be an alternative to PDMS. However, there are some limitations of the FEPM elastomers. They are 100 times less gas permeable than PDMS and thus perfusion culture is required to maintain cellular viability. On the other hand, high permeation of water vapor of PDMS devices causes evaporation and osmolarity shifts that affect cell growth and development [25,46]. The FEPM devices may mitigate this problem because of low permeability of water vapor [47]. Because FEPM has hydrophobicity and low gas permeability, air bubbles may be generated inside channels when solutions are introduced. These bubbles can be removed by incubating the devices with culture medium in an incubator before cell seeding. Another limitation is that a large vacuum compression molding machine and precision-metal molds are required to fabricate channel layers. This is not user friendly; however, this may be suitable for mass production because the vacuum compression molding machine is commonly used for mass production of rubber products, whereas PDMS is incompatible with mass production due to the low speed of fabrication processes. Furthermore, this method can produce 100 µm features (Figure S5) although we used millimeter channels in this study.

In summary, we developed a novel microfluidic culture device using the FEPM elastomer with thin ECM membranes. Since this elastomer is resistant to drug absorption, the FEPM devices can be used to test the effects of drugs. This microfluidic device permits the formation of a tissue–tissue interface on a thin ECM membrane and the application of mechanical forces including cyclic strain and fluid shear stress. This device also allows real-time imaging of cellular responses. Therefore, this device could be a useful platform to construct organs-on-chips for drug discovery and development.

## Supplementary Materials

The following are available online at https://www.mdpi.com/2072-666X/10/11/793/s1: Video S1: Cells cultured in the FEPM microfluidic device while applying cyclic strain. Figure S1: Design of the FEPM channel layer. Figure S2: Evaluation of fluorescence intensity of the FEPM device containing a rhodamine B solution. Figure S3: Images of HUVECs cultured on a cell culture plate. Figure S4: Evaluation of cellular viability after 24 h treatment of mitomycin C. Figure S5: Images of FEPM layers containing small features.
