# Supplementary material for: Tetrafluoroethylene-Propylene Elastomer for Fabrication of Microfluidic Organs-on-Chips Resistant to Drug Absorption
Source: Micromachines (Basel). 2019 Nov 19;10(11):793. doi: 10.3390/mi10110793 (PMC6915658; doi:10.3390/mi10110793)
Supplement: Supplementary file 1 [file micromachines-10-00793-s001.zip › micromachines-591681-supplementary.pdf]

micromachines

Supporting Information

Tetrafluoroethylene-Propylene Elastomer for Fabrication of Microfluidic Organs-on-Chips Resistant to Drug Absorption

Emi Sano [1,†], Chihiro Mori [1,†], Naoki Matsuoka [2], Yuka Ozaki [1], Keisuke Yagi [2], Aya Wada [2], Koichi Tashima [2], Shinsuke Yamasaki [2], Kana Tanabe [2], Kayo Yano [1] and Yu-suke Torisawa [1,3,*]

1 Department of Micro Engineering, Kyoto University, Kyoto 615-8540, Japan; e.sano1017@gmail.com (E.S.); mori.chihiro.5u@kyoto-u.ac.jp (C.M.); ozaki.yuka.6z@kyoto-u.ac.jp (Y.O.); yano.kayo.7z@kyoto-u.ac.jp (K.Y.)
2 AGC Inc, Tokyo 100-8405, Japan; naoki-matsuoka@agc.com (N.M.); keisuke-yagi@agc.com (K.Y.); aya-wada@agc.com (A.W.); kouichi-tashima@agc.com (K.T.); shinsuke.yamasaki@agc.com (S.Y.); kana-.tanabe@agc.com (K.T.)
3 Hakubi Center for Advanced Research, Kyoto University, Kyoto 615-8540, Japan
* Correspondence: torisawa.yusuke.6z@kyoto-u.ac.jp; Tel.: +81-75-383-3701
† These authors contributed equally to this work.

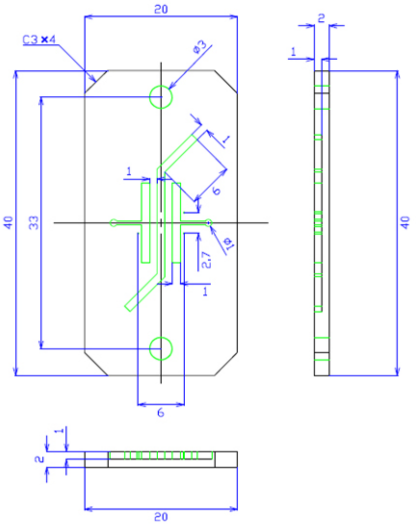

Supplementary Fig. S1. Design of the FEPM channel layer. All dimensions in millimeters.

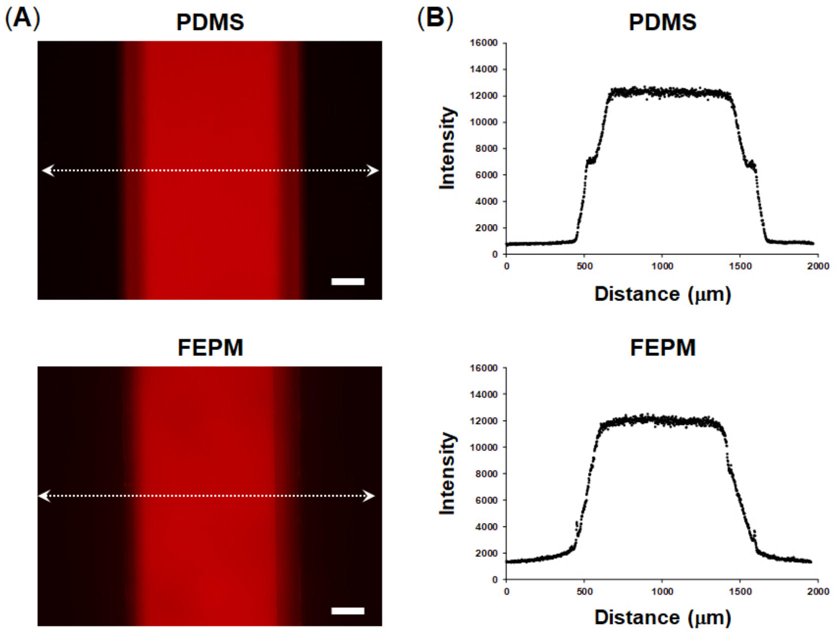

Supplementary Fig. S2. (A) Fluorescence images of a solution containing 10 μM Rhodamine B inside PDMS and FEPM channels with an exposure time of 5 ms. Scale bars, 200 μm. (B) Line profiles of the fluorescence images shown in (A) (indicated by white dotted lines). (n=3)

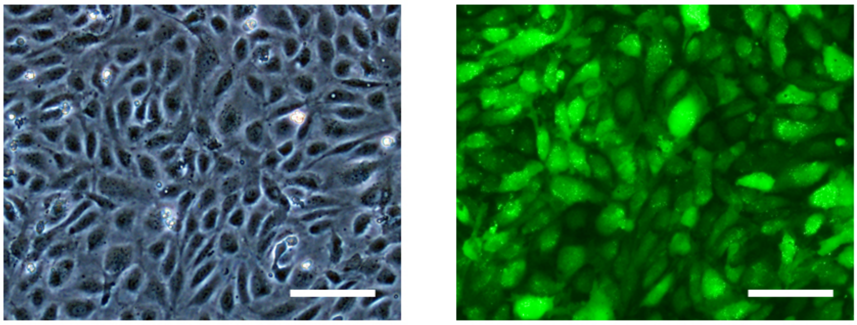

Supplementary Fig. S3. Optical (left) and fluorescence (right) images of GFP-expressing HUVECs cultured on a cell culture plate for 7 days. (n=3) Scale bars, 100 μm.

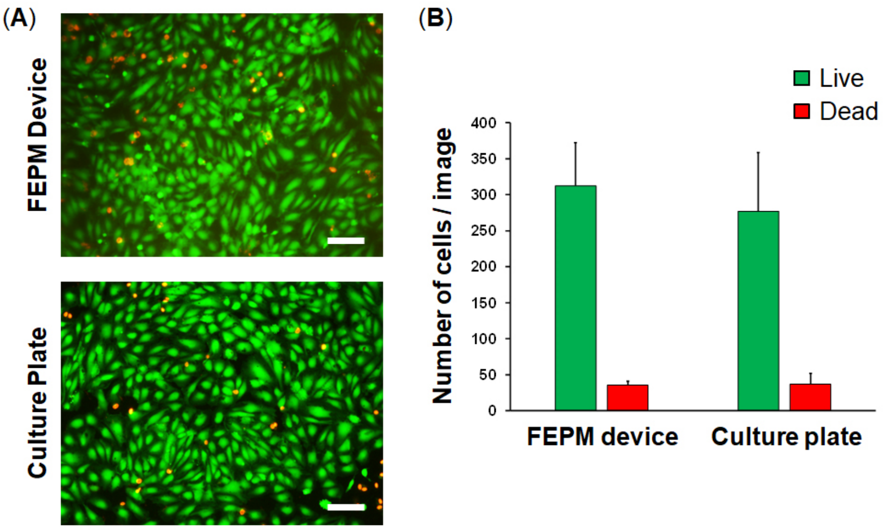

Supplementary Fig. S4. Cellular viability after 24 hours treatment of mitomycin C. (A) Fluorescence images of lived/dead strained HUVECs in the FEPM device and in a 96-well culture plate 24 hours after incubation with mitomycin C (10 μg mL^{-1}). Scale bars, 100 μm. (B) The average number of live and dead cells in each image area (ca. 0.58 mm^2) evaluated using 3 different observation areas from 3 independent experiments.

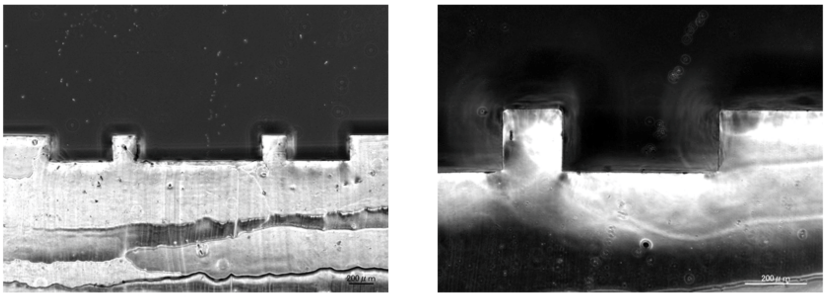

Supplementary Fig. S5. Cross-sectional images of FEPM layers consisting of small channels. The cross-sectional sizes of the channels are1 mm (width) x 200 μm (height) for the central channel and 500 μm (with) x 200 μm (height) for the side channels. The gaps between the microchannels are 100 μm. Scale bars, 200 μm.
